# A Computed Tomography Radiomics-Based Prediction Model on Interstitial Lung Disease in Anti-MDA5-Positive Dermatomyositis

**DOI:** 10.3389/fmed.2021.768052

**Published:** 2021-11-29

**Authors:** Wenwen Xu, Wanlong Wu, Yu Zheng, Zhiwei Chen, Xinwei Tao, Danting Zhang, Jiangfeng Zhao, Kaiwen Wang, Bingpeng Guo, Qun Luo, Qian Han, Yan Zhou, Shuang Ye

**Affiliations:** ^1^Department of Rheumatology, Renji Hospital, Shanghai Jiao Tong University School of Medicine, Shanghai, China; ^2^Department of Pulmonology, Renji Hospital, Shanghai Jiao Tong University School of Medicine, Shanghai, China; ^3^CT Scientific Collaboration, Siemens Healthineers, Shanghai, China; ^4^State Key Laboratory of Respiratory Disease, National Clinical Center for Respiratory Disease, Guangzhou Institute of Respiratory Health, The First Affiliated Hospital of Guangzhou Medical University, Guangzhou, China; ^5^Department of Radiology, Renji Hospital, Shanghai Jiao Tong University School of Medicine, Shanghai, China

**Keywords:** anti-melanoma differentiation-associated gene 5, dermatomyositis, interstitial lung disease, prognosis, radiomics

## Abstract

**Objectives:** Anti-melanoma differentiation-associated gene 5-positive dermatomyositis-associated interstitial lung disease (MDA5^+^ DM-ILD) is a life-threatening disease. The current study aimed to quantitatively assess the pulmonary high-resolution computed tomography (HRCT) images of MDA5^+^ DM-ILD by applying the radiomics approach and establish a multidimensional risk prediction model for the 6-month mortality.

**Methods:** This retrospective study was conducted in 228 patients from two centers, namely, a derivation cohort and a longitudinal internal validation cohort in Renji Hospital, as well as an external validation cohort in Guangzhou. The derivation cohort was randomly divided into training and testing sets. The primary outcome was 6-month all-cause mortality since the time of admission. Baseline pulmonary HRCT images were quantitatively analyzed by radiomics approach, and a radiomic score (Rad-score) was generated. Clinical predictors selected by univariable Cox regression were further incorporated with the Rad-score, to enhance the prediction performance of the final model (Rad-score plus model). In parallel, an idiopathic pulmonary fibrosis (IPF)-based visual CT score and ILD-GAP score were calculated as comparators.

**Results:** The Rad-score was significantly associated with the 6-month mortality, outperformed the traditional visual score and ILD-GAP score. The Rad-score plus model was successfully developed to predict the 6-month mortality, with C-index values of 0.88 [95% confidence interval (CI), 0.79–0.96] in the training set (*n* = 121), 0.88 (95%CI, 0.71–1.0) in the testing set (*n* = 31), 0.83 (95%CI, 0.68–0.98) in the internal validation cohort (*n* = 44), and 0.84 (95%CI, 0.64–1.0) in the external validation cohort (*n* = 32).

**Conclusions:** The radiomic feature was an independent and reliable prognostic predictor for MDA5^+^ DM-ILD.

## Introduction

Anti-melanoma differentiation-associated gene 5-positive dermatomyositis (MDA5^+^ DM) is a distinct subtype of dermatomyositis (DM) predominantly reported in East Asia, characterized by pathognomonic rashes, none or mild myositis, and, notably, rapidly progressive interstitial lung disease (ILD). Recent study had reported three subgroups of MDA5^+^ DM with different prognoses. Patients with rapidly progressive ILD had a very high mortality rate, compared to those with frequent signs of skin vasculopathy and with frequent arthralgia or arthritis (1). The overall prognosis of MDA5^+^ DM-ILD is poor with most fatalities occurring within the first half year since disease onset. A gravely 6-month mortality rate of around 50% was reported despite the intensive immunosuppressive therapy (2–5).

As a mainstream imaging tool for identifying ILD, pulmonary high-resolution computed tomography (HRCT) is also crucial in the prognostic prediction. Moreover, semi-quantitative HRCT score has been applied as a prognostic prediction measurement in MDA5^+^ DM-ILD (6, 7). However, currently available HRCT scoring methods are observer dependent, with considerable inter-observer bias and initially derived for idiopathic pulmonary fibrosis (IPF) (8–10). When referring to MDA5^+^ DM-ILD with a much more aggressive clinical course, the applicability of the scoring system is controversial (11). Therefore, a measurable and quantitative method to assess severity and predict prognosis of MDA5^+^ DM-ILD is urgently needed. The technological advance of “radiomics” has provided a promising solution for HRCT evaluation in a more comprehensive and objective perspective. The technological advance of “radiomics” is a conversion of digital medical images into mineable high-dimensional data. It may provide a promising solution for HRCT evaluation in a more comprehensive and objective perspective. Since the radiographic images contain information that reflects underlying pathophysiology, Radiomics could improve predictive or prognostic accuracy potentially. In the area of oncology, radiomics has enabled the non-invasive profiling of tumor heterogeneity and direct estimation of prognosis by high-throughput extraction of quantitative descriptors from radiographic images (12, 13). To the best of our knowledge, there was no radiomics study focusing on MDA5^+^ DM-ILD. Therefore, the current study aimed to quantitatively assess MDA5^+^ DM-ILD by applying the radiomics approach and further establish an applicable multidimensional risk prediction model by incorporating clinical factors for 6-month mortality to improve the prognosis of this disease.

## Materials and Methods

### Patients

The study population consisted of hospitalized patients from two centers: A derivation cohort from April 2014 to October 2019 and a longitudinal internal validation cohort from November 2019 to May 2020 were recruited in Renji Hospital (Shanghai). However, an external validation cohort from Guangzhou Institute of Respiratory Health was constructed during the same time period. Ethical approval was obtained for this retrospective analysis, and the need to obtain informed consent was waived for both institutions. Eligible patients for this study initially fulfilled Bohan and Peter's criteria for DM or Sontheimer's criteria for clinically amyopathic DM on admission (14, 15) and were retrospectively confirmed by the 239th European NeuroMuscular Center (ENMC) classification criteria for DM (16). All patients presented with imaging-confirmed ILD and positive anti-MDA5 antibody. The anti-MDA5 antibody was detected by immunoblotting assay (Euroimmun, Germany) and confirmed by enzyme-linked immunosorbent assay (Supplementary Material). ILD course was defined as time from the first abnormal pulmonary CT, which revealed ILD changes to admission. Patients with ILD course > 3 months or coexisting malignancy (within 3 years) or preexisting chronic obstructive pulmonary disease were excluded. The primary outcome was the 6-month all-cause mortality since the time of admission.

**Figure 1:**
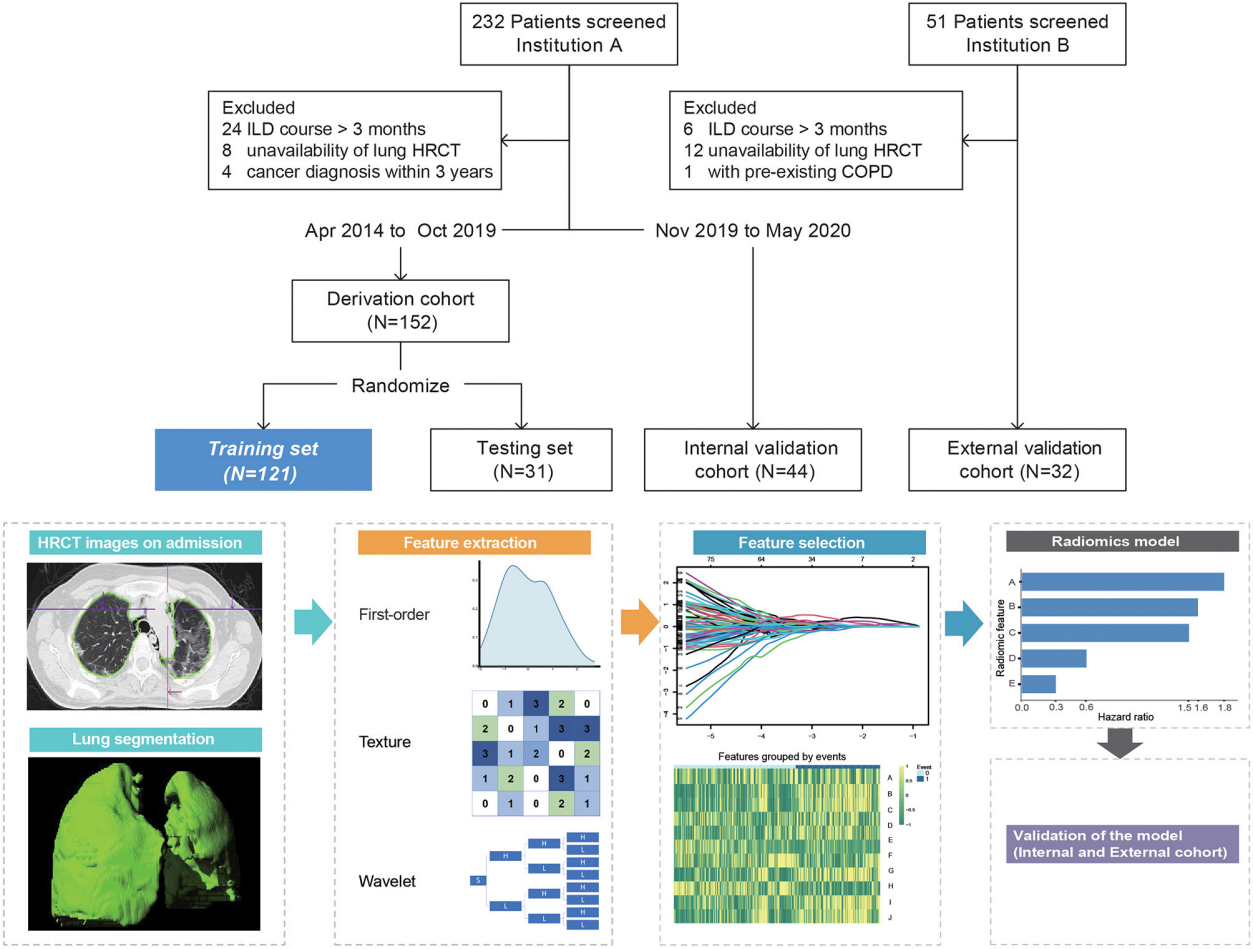
Overview of patient selection and workflow of radiomics model construction. Radiomic feature A = Original_Shape_Flatness (HR 1.82, 95%CI 1.28–2.59, *p* < 0.001), B = Wavelet.LLL_Glcm_MCC (HR 1.59, 95%CI 0.97–2.6, *p* = 0.068), C = Wavelet.LLH_Glcm_Ldmn (HR 1.5, 95%CI 1.02–2.22, *p* = 0.04), D = Wavelet.HLL_Glszm_LargeAreaHighGrayLevelEmphasis (HR 0.58, 95%CI 0.38–0.89, *p* < 0.001), and E = Wavelet.LLL_Firstorder_Skewness (HR 0.30, 95%CI 0.18–0.52, *p* < 0.001). ILD course, time from the first abnormal pulmonary CT that revealed ILD changes to admission; HRCT, high-resolution computed tomography; COPD, chronic obstructive pulmonary disease.

The derivation cohort was randomly divided into the training (*n* = 121) and testing (*n* = 31) sets. The internal and external validation cohorts comprised 44 and 32 patients, respectively (Figure 1).

### Radiomics Analysis of HRCT Images

Patients underwent non-contrast pulmonary HRCT within a week of admission (median, 2 days; range, 1–6 days) using a multidetector CT scanner. CT slice thickness was 1.0–1.5 mm at 10-mm interval with standard reconstruction. Radiomics analysis was performed using a dedicated software (Radiomics, syngo.via Frontier 1.2.2, Siemens Healthineers, Forchheim, Germany). This platform integrated an open-source library named “PyRadiomics” (version 2.0.1). First, the Digital Imaging and Communications in Medicine files of CT images were imported; segmentation of the volumetric lung was performed automatically with deep learning algorithm and adjusted manually by ZC and YZ (both with more than 5 years' experience in chest CT imaging). The medical image series were resampled to 1 1 1 mm voxel size with B-spline interpolator before subsequent feature extraction steps. Bin width was set at 25 to create histograms for discretization of the image gray levels. The extracted features are as follows: (i) 18 first-order features, which describe the distribution of voxel intensities in region of interest (ROI); (ii) 18 shape and size features, which include descriptors of 2D and 3D size and shape of ROI; (iii) 75 texture features, describing patterns or the spatial distribution of voxel intensities. In addition to the original images, wavelet-transformed images were generated using eight different high- to low-frequency band combinations according to three directions.

Thus, a total of 855 features, namely, 111 from the original image and 744 from the wavelet-transformed images, were outputted. Then, the intra- and inter-observer reliabilities were evaluated using Spearman's correlation coefficients in derivation cohort; features with rho>0.8 were included for further analysis.

### Radiomics Model Construction and Radiomic Score (Rad-Score) Generation

The radiomics model was derived from the training set and tuned in the testing set. The least absolute shrinkage and selection operator (LASSO) and Cox regression model was used to select features, with penalty parameter tuning conducted by ten-fold cross-validation (17). Then, optimal features for radiomics model were determined by backward stepdown analysis with Akaike's information criterion (AIC) applied as the stopping rule.

Radiomic score for each patient was calculated from a linear combination of selected radiomic features weighted by respective coefficients.

### Visual HRCT Scoring and ILD-GAP Model

All CT images were reviewed by ZC and YZ who were blinded to patients' outcome. Inter-observer variability was evaluated by intraclass correlation coefficient (ICC). The results were agreed upon by consensus between the two observers. HRCT findings were graded on a scale of 1–6 based on the classification system that corresponded to consecutive pathologic phases as previously reported (8): 1, normal attenuation; 2, ground-glass attenuation (GGA); 3, consolidation; 4, GGA associated with traction bronchiolectasis or bronchiectasis; 5, consolidation associated with traction bronchiolectasis or bronchiectasis; and 6, honeycombing.

The lungs were divided into six zones (upper, middle, and lower on both sides); each zone was evaluated separately. The score was based on the percentage of the abnormal lung parenchyma and was estimated to the nearest 5% of parenchymal involvement. The overall CT visual score was calculated by adding the average score of six zones (6).

In addition, an ILD-GAP score without lung imaging information was also calculated in our analysis as a comparator, which was modified from the IPF-derived GAP staging system and had been validated in some non-IPF ILD cohorts for prognosis prediction (18, 19).

Finally, a subgroup analysis was performed to show in which cases the radiomics model outperformed the visual scoring model.

### Statistical Analysis

The differences of clinical parameters between the derivation and internal validation and external validation cohorts were assessed by the Mann–Whitney *U*-test, chi-square test, or Fisher exact test, as appropriate. *Post hoc* comparisons were made with Bonferroni's correction for adjustments of multiple comparisons. The optimal cutoff values for significant continuous variables were identified by X-tile (20). The association between Rad-score and outcome was assessed by Kaplan–Meier survival plot and log-rank test.

An improved multidimensional prediction model based upon Rad-score (Rad-score plus model) was constructed by integrating clinically significant predictors from univariable analysis into the multivariable Cox proportional hazards model (backward stepdown selection with AIC).

Correlation analysis between visual score, Rad-score, and other variables were applied with Spearman's coefficients. Model discrimination was quantified by the Harrell concordance index (C-index) with 95%CI. The change in overall log-likelihood ratio was used to assess the increase in predictive power. Additionally, the model calibration was assessed using the calibration curve, Greenwood–Nam–D'Agostino (GND) test, and Brier score (13, 21, 22). A decision curve analysis was built to determine and compare the clinical usefulness of each model (23). Significance was defined as *p*-value <0.05.

Statistical analyses were performed on SPSS software version 25 (IBM Corp., Armonk, NY, United States) and R software version 3.6.1. All R codes used were available at GitHub (https://github.com/tomato08217/MDA-ILD5).

## Results

The all-cause 6-month mortality was 62/152 (40.8%), 17/44 (38.6%), and 14/32 (43.8%) for derivation, internal validation, and external validation cohorts, respectively. Table 1 presents the comparable clinical characteristics of the three cohorts in detail. Comparisons between the training and testing sets of the derivation cohort are also listed in Supplementary Table 1. A total of 17.8% patients lacked the data of the forced vital capacity percentage of predicted (FVC%), since they were in severe condition unable to complete either routine or bedside spirometry. For the remaining patients, the optimal cutoff values calculated for FVC% were 49.7% (rounding to 50%). Therefore, we classified FVC% into three categories: FVC% ≥ 50%, FVC% <50%, and unable to perform pulmonary function tests.

**Table 1:** Comparison of baseline clinical features and outcomes between three cohorts.

**Characteristics**	**Derivation cohort (*n* = 152)**	**Internal validation cohort (*n* = 44)**	**External validation cohort (*n* = 32)**	***p*-value**	**Missing**
6-month mortality	62 (40.8%)	17 (38.6%)	14 (43.8%)	0.91	0
**Demographic**					
Male	54 (35.5%)	17 (38.6%)	14 (43.8%)	0.67	0
Age, years	50 [42–58]	52 [47–57]	52 [45–56]	0.66	0
DM course^*^, month	2 [2–4]	2 [1–3]	2 [1–3]	0.39	0
ILD-course^†^, week	4 [2–8]	4 [2–7]	4 [2–8]	0.98	0
**Pulmonary function**					
Three-category FVC%				0.12	7
FVC% ≥50%	83 (54.6%)	28 (65.1%)	16 (61.5%)		
FVC% <50%	42 (27.6%)	8 (18.6%)	2 (7.69%)		
Unable to perform PFT^‡^	27 (17.8%)	7 (16.3%)	8 (30.8%)		
PaO_2_/FiO_2_ <200mmHg	22 (14.5%)	6 (13.6%)	5 (15.6%)	0.96	0
**Laboratory data**					
Serum ferritin, ng/mL	1,037 [395–1,850]	1,203 [560–2,080]	1,699 [906–2,000]	0.22	11
LDH, U/L	330 [257–456]	307 [249–388]	274 [230–408]	0.20	3
Lymphocyte, 10^9^/L	0.7 [0.5–1.1]	0.7 [0.5–0.9]	0.6 [0.4–0.9]	0.43	0
Cytopenia	27 (17.8%)	6 (13.6%)	7 (21.9%)	0.64	0
CKmax, U/L	107 [41–306]	94 [45–216]	59 [33–123]	0.07	0
ALT, U/L	63 [37–106]	63 [49–140]	37 [24–55]	<0.001	0
AST, U/L	60 [34–105]	68 [47–114]	46 [32–56]	0.01	0
Anti-Ro52 Ab positive	91 (59.9%)	34 (77.3%)	23 (71.9%)	0.07	0
Anti-MDA5 Ab titer, RU/mL	179.8 [148.7–228.2]	192.5 [162.9–226.2]	189.7 [152.5–217.6]	0.33	9
**Treatment**					
Max dosage of MP, mg/d	120 [80–230]	80 [60–160]	500 [250–500]	<0.001	0
Steroid pulse therapy	17 (11.2%)	1 (2.27%)	19 (59.4%)	<0.001	0
**Exposure to IS^§^**					
1 IS	54 (35.5%)	27 (61.4%)	3 (9.38%)	<0.001	0
≥2 IS	84 (55.3%)	17 (38.6%)	29 (90.6%)	<0.001	0
Exposure to pirfenidone or nintedanib	69 (45.4%)	14 (31.8%)	16 (50.0%)	0.2	0

*Data are presented as median [IQR] for continuous variables and number (frequency) (%) for categorical variables*.

* *DM course, time from the first symptom of dermatomyositis (DM) to admission*.

† *ILD-course, time from the first abnormal pulmonary CT which revealed ILD changes to admission*.

‡ *Unable to perform PFT, referred to those patients in severe condition that unable to complete either routine or bedside spirometry*.

§ *IS, immunosuppressant drugs, including cyclophosphamide, cyclosporine, tacrolimus, mycophenolate mofetil, tofacitinib, rituximab, basiliximab, and tocilizumab*.

*ILD, interstitial lung disease; FVC%, forced vital capacity percentage of predicted; PFT, pulmonary function test; PaO2/FiO2, arterial oxygen/fraction of inspiration oxygen; LDH, lactate dehydrogenase; CKmax, maximum creatine kinase from disease onset to admission; ALT, alanine transaminase; AST, aspartate transaminase; MDA5, melanoma differentiation-associated protein 5; Ab, antibody; MP, methylprednisolone*.

Notably, the treatment regimens differed between the two centers, with significantly higher intensity of glucocorticoid and immunosuppressant exposure in the external validation cohort (Table 1). And, in the derivation cohort, the deceased received significantly higher dose of glucocorticoid than survivors, while the intensity of immunosuppressant use was comparable between the two groups (Table 2).

**Table 2:** Univariable Cox regression results of baseline clinical characteristics and treatment between the survivors and deceased in the derivation cohort.

**Characteristics**	**Derivation cohort (*n* = 152)**	**Survivors** **(*n* = 90)**	**Deceased (*n* = 62)**	***p*-value**
**Demographic**				
Male sex	54 (35.5%)	31 (34.4%)	23 (37.1%)	0.92
Age, years	50 [42–58]	48 [39–54]	52 [45–62]	0.001
DM course^*^, month	2 [2–4]	3 [2–5]	2 [1–3]	0.04
ILD-course^†^, week	4 [2–8]	4 [2–8]	4 [2–8]	0.192
**Extrapulmonary symptoms**				
Fever	99 (65.1%)	54 (60%)	45 (72.6%)	0.36
Arthralgia	77 (50.7%)	54 (60%)	23 (37.1%)	0.02
Pharyngalgia	21 (13.8%)	12 (13.3%)	9 (14.5%)	0.98
Heliotrope sign	130 (85.5%)	75 (83.3%)	55 (88.7%)	0.34
Gottron sign	126 (82.9%)	72 (80%)	54 (87.1%)	0.40
Skin ulcer	27 (17.8%)	19 (21.1%)	8 (12.9%)	0.22
Dysphagia	22 (14.5%)	13 (14.4%)	9 (14.5%)	0.78
**Pulmonary function**				
FVC%	60.8 [45.9–72.5]	65.7 [53.2–76.8]	47.8 [37.3–61.1]	0.001
Unable to perform PFT^‡^	27 (17.8%)	1 (1.1%)	26 (41.9%)	0.001
PaO_2_/FiO_2_, mmHg	334 [257–389]	371 [310–410]	258 [158–326]	0.001
**Laboratory data**				
Serum ferritin, ng/mL	1,037 [395–1,850]	811 [339–1,380]	1,415 [557–3,318]	0.001
LDH, U/L	330 [257–456]	292 [226–390]	473 [342–702]	0.001
CRP, mg/L	4.0 [0.2–10.6]	2.5 [0.1–7.2]	6.0 [0.7–18.2]	0.001
ESR, mm/H	32 [14–47]	30 [14–48]	33 [14–46]	0.92
Lymphocyte, 10^9^/L	0.7 [0.5–1.1]	0.8 [0.5–1.2]	0.6 [0.4–0.8]	0.002
Cytopenia	27 (17.8%)	15 (16.7%)	12 (19.4%)	0.42
Ckmax, U/L	107 [41–306]	78 [35–264]	166 [53–374]	0.28
ALT, U/L	62 [37–106]	50 [31–90]	77 [50–122]	0.63
AST, U/L	60 [34–105]	44 [28–79]	78 [48–134]	0.55
Anti-Ro52 Ab positive	91 (59.9%)	50 (55.6%)	41 (66.1%)	0.78
Anti-MDA5 Ab titer, RU/mL	179.8 [148.7–228.2]	178.3 [144.6–219.2]	185.2 [155.0–238.9]	0.35
**Treatment**				
Max dosage of MP, mg/d	120 [80–230]	80 [50–160]	160 [120–240]	<0.001
Steroid pulse therapy	17 (11.2%)	5 (5.6%)	12 (19.4%)	0.002
**Exposure to IS** ^§^				
1 IS	54 (35.5%)	37 (41.1)	17 (27.4)	0.09
≥2 IS	84 (55.3%)	49 (54.4)	35 (56.5)	0.89
Exposure to pirfenidone or nintedanib	69 (45.4%)	42 (46.7)	27 (43.5)	0.62

*Data are presented as median [IQR] for continuous variables and number (frequency) (%) for categorical variables*.

* *DM course, time from the first symptom of dermatomyositis (DM) to admission*.

† *ILD-course, time from the first abnormal pulmonary CT which revealed ILD changes to admission*.

‡ *Unable to perform PFT, referred to those patients in severe condition that unable to complete either routine or bedside spirometry*.

§ *IS, immunosuppressant drugs, including cyclophosphamide, cyclosporine, tacrolimus, mycophenolate mofetil, tofacitinib, rituximab, basiliximab, and tocilizumab*.

*ILD, interstitial lung disease; FVC%, forced vital capacity percentage of predicted; PFT, pulmonary function test; PaO2/FiO2, arterial oxygen/fraction of inspiration oxygen; CRP, C-reactive protein; ESR, erythrocyte sedimentation rate; LDH, lactate dehydrogenase; CKmax, maximum creatine kinase from disease onset to admission; ALT, alanine transaminase; AST, aspartate transaminase; MDA5, melanoma differentiation-associated protein 5; Ab, antibody; MP, methylprednisolone*.

### Radiomics Model and Rad-Score

Eight hundred and twenty-five features remained after reproducibility analysis. Five radiomic features were finally selected for modeling as follows: Original_Shape_Flatness (A), Wavelet.LLL_Glcm_MCC (B), Wavelet.LLH_Glcm_Ldmn (C), Wavelet.HLL_Glszm_LargeAreaHighGrayLevelEmphasis (D), and Wavelet.LLL_Firstorder_Skewness (E).

The contribution of the selected parameters with their regression coefficients was presented in the form of a histogram in Figure 1. Then, the Rad-score was computed using the following formula: A*(0.599) +B*(0.461) +C*(0.408) +D*(−0.543) +E*(−1.19). The C-index values of the radiomics model were 0.83 (95%CI, 0.74–0.91), 0.82 (95%CI, 0.65–0.99), 0.78 (95%CI, 0.64–0.93), and 0.76 (95%CI, 0.59–0.92) for the training, testing, internal validation, and external validation sets, respectively. The GND test showed good calibration abilities in the four datasets. The prediction errors were low, attested by Brier score ranging from 0.12 to 0.19.

**Figure 2:**
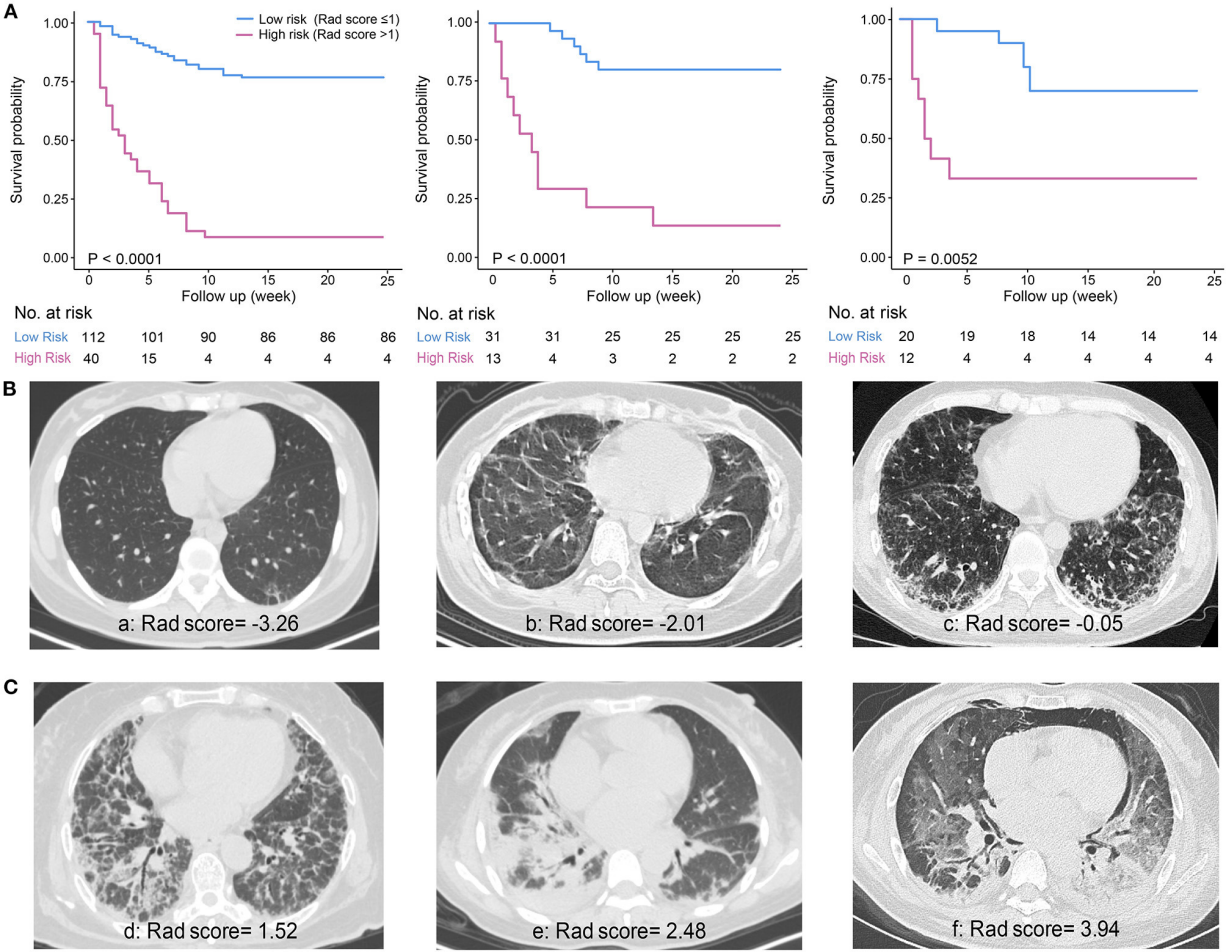
Survival curves of the Rad-score and representative CT images. **(A)** Significant difference between low-risk (Rad-score ≤ 1) and high-risk (Rad-score > 1) groups was shown in the derivation cohort (*n* = 152, left) and verified in the internal validation (*n* = 44, middle) and external validation cohort (*n* = 32, right). **(B)** Representative patients' CT images of low-risk group. a: A 32-year-old female patient with discrete subpleural reticulation and ground-glass attenuation (GGA); b: a 23-year-old female patient with typical subpleural lines and GGA; c: a 39-year-old male patient showing a number of GGA and reticulation with some basal consolidation. **(C)** Representative patients' CT images of high-risk group. d: A 62-year-old female patient with a large amount of reticulation pattern accompanied by GGA and consolidation; e: a 47-year-old female patient with predominant consolidation; f: a 41-year-old female patient with diffuse GGA and consolidation accompanied by pneumomediastinum.

The optimal cutoff value for Rad-score was determined as 1, dividing patients into the high-risk group (Rad-score > 1) and low-risk group (Rad-score ≤ 1) with distinct 6-month mortality (Figure 2A). The representative patients' CT images of the two groups are presented in Figures 2B,C, respectively, with corresponding Rad-scores.

### Rad-Score Plus Model

The univariable Cox regression results of baseline clinical characteristics and treatment between the survivors and deceased in the derivation cohort are presented in Table 2. Then, 10 candidate predictors, i.e., with significant differences in univariable analyses, were incorporated into a multivariable Cox proportional hazards model with Rad-score, indicated as follows: age on admission, course of DM, arthralgia, three-category FVC% (FVC% ≥ 50%, FVC% <50%, and unable to perform pulmonary function tests), arterial oxygen/fraction of inspiration oxygen (PaO_2_/FiO_2_), lactate dehydrogenase (LDH), serum ferritin (ng/mL), C-reactive protein, lymphocyte, and maximum dosage of methylprednisolone (Table 3).

**Table 3:** Results of univariable and multivariable Cox regression.

	**Univariable analysis**		**Multivariable analysis**	
	**HR (95%CI)**	***p*-value**	**HR (95%CI)**	***p*-value**
Rad-score	2.57 [2.09–3.17]	<0.001	1.74 [1.28–2.36]	<0.001
Age, years	1.04 [1.02–1.07]	<0.001	1.03 [1.01–1.06]	0.02
DM course^*^, month	0.83 [0.70–0.99]	0.036		
Arthralgia	0.48 [0.28–0.80]	0.019		
**Three-category FVC%**				
FVC% ≥ 50%	Reference		Reference	
FVC% <5 0%	4.04 [2.06–7.90]	<0.001	2.78 [1.25–6.22]	0.01
Unable to perform PFT^†^	22.3 [11.2–44.4]	<0.001	9.47 [3.41–26.3]	<0.001
PaO_2_/FiO_2_, mmHg	0.989 [0.986,0.992]	<0.001		
Serum ferritin, ng/mL	1.002 [1.001,1.003]	<0.001		
LDH, U/L	1.004 [1.003–1.005]	<0.001		
CRP, mg/L	1.04 [1.02–1.05]	0.002		
Lymphocyte, 10^9^/L	0.30 [0.14–0.65]	<0.001		
Max dosage of MP, mg/d	1.004 [1.002–1.005]	<0.001		

* *DM course, time from the first symptom of dermatomyositis (DM) to admission*.

† *Unable to perform PFT, referred to those patients in severe condition that unable to complete either routine or bedside spirometry*.

*HR, hazard ratio; 95%CI, 95% confidence interval; FVC%, forced vital capacity percentage of predicted; PFT, pulmonary function test; PaO2/FiO2, arterial oxygen/fraction of inspiration oxygen; CRP, C-reactive protein; LDH, lactate dehydrogenase; MP, methylprednisolone*.

The optimal combination obtained by the backward stepwise algorithm with minimum AIC was as follows: Rad-score, FVC% (three categories), and age (on admission). The respective hazard ratios and β-regression coefficients are presented in Table 4. The final Rad-score plus model was developed to predict the 6-month mortality with C-index values of 0.88 (95%CI, 0.79–0.96) in the training set, 0.88 (95%CI, 0.71–1.0) in the testing set, 0.83 (95%CI, 0.68–0.98) in the internal validation cohort, and 0.84 (95%CI, 0.64–1.0) in the external validation cohort, respectively. Nomogram was presented online to provide individualized risk estimates (https://mda5-ild.shinyapps.io/MDA_ILD5_test/).

**Table 4:** Results of multivariable Cox regression analysis based on Rad-score.

**Rad-score plus model**	**HR [95%CI]**	***p*-value**	**β-coefficient**
**Rad-score+FVC%+age**			
Rad-score	1.74 [1.28-2.36]	<0.001	0.55
Three-category FVC%			
FVC% ≥ 50%	reference		
FVC% <50%	2.78 [1.25-6.22]	0.01	1.02
Unable to perform PFT^*^	9.47 [3.41-26.3]	<0.001	2.25
Age (year)	1.03 [1.01-1.06]	0.02	0.03

* *Unable to perform PFT, referred to those patients with severe condition who were unable to complete either routine or bedside spirometry*.

*HR, hazard ratio; 95%CI, 95% confidence interval; FVC%, the forced vital capacity percentage of predicted; age, age on admission; PFT, pulmonary function test*.

### Visual Score and ILD-GAP Model

The agreement of visual CT score between two readers was good with ICC of 0.821 (95%CI, 0.711–0.898). The visual score model yielded the C-index values of 0.75 (95%CI, 0.66–0.84) for the training set, 0.81 (95%CI, 0.65–0.98) for the testing set, and 0.79 (95%CI, 0.65–0.94) for the internal validation cohort, when predicting the 6-month mortality of MDA5^+^ DM-ILD. However, the discriminative power of visual score dramatically declined in the external validation cohort with the C-index of 0.66 (95%CI, 0.49–0.82) and poor calibration ability. However, the C-index values of ILD-GAP model were relatively even in each dataset (see Table 5 for details).

Table 5Comparison of the predictive performance of each model.

**Table:** 

	**Training set (*****n*** **= 121)**			**Testing set (*****n*** **= 31)**			**AIC**
	**C-index [95%CI]**	**GND**	**Brier score**	**C-index [95%CI]**	**GND**	**Brier score**	
ILD-GAP model	0.73 [0.67–0.78]	0.56	0.17	0.78 [0.67–0.88]	0.37	0.16	438.68
Visual score model	0.75 [0.66–0.84]	0.06	0.18	0.81 [0.65–0.98]	0.21	0.16	414.08
Radiomics model	0.83 [0.74–0.91]	0.37	0.12	0.82 [0.65–0.99]	0.26	0.15	371.05
**Rad-score plus model**							
Rad-score + FVC% + age	0.88 [0.79–0.96]	0.69	0.11	0.88 [0.71–1.0]	0.45	0.11	355.84

**Table:** 

	**Internal validation cohort (*****n*** **= 44)**			**External validation cohort (*****n*** **= 32)**		
	**C-index [95%CI]**	**GND**	**Brier score**	**C-index [95%CI]**	**GND**	**Brier score**
ILD-GAP model	0.76 [0.65–0.87]	0.36	0.13	0.74 [0.59–0.9]	0.52	0.19
Visual score model	0.79 [0.65–0.94]	0.36	0.14	0.66 [0.49–0.82]	0.03	0.22
Radiomics model	0.78 [0.64–0.93]	0.59	0.15	0.76 [0.59–0.92]	0.41	0.19
**Rad-score plus model**						
Rad-score + FVC% + age	0.83 [0.68–0.98]	0.54	0.13	0.84 [0.64–1.0]	0.34	0.11

*ILD, interstitial lung disease; C-index, concordance index; 95%CI, 95% confidence interval; GND, Greenwood-Nam-D'Agostino test; AIC, Akaike's information criterion evaluating the risk of overfitting; FVC%, forced vital capacity percentage of predicted; age, age on admission*.

### Comparison of the Predictive Performance of Each Model

**Figure 3:**
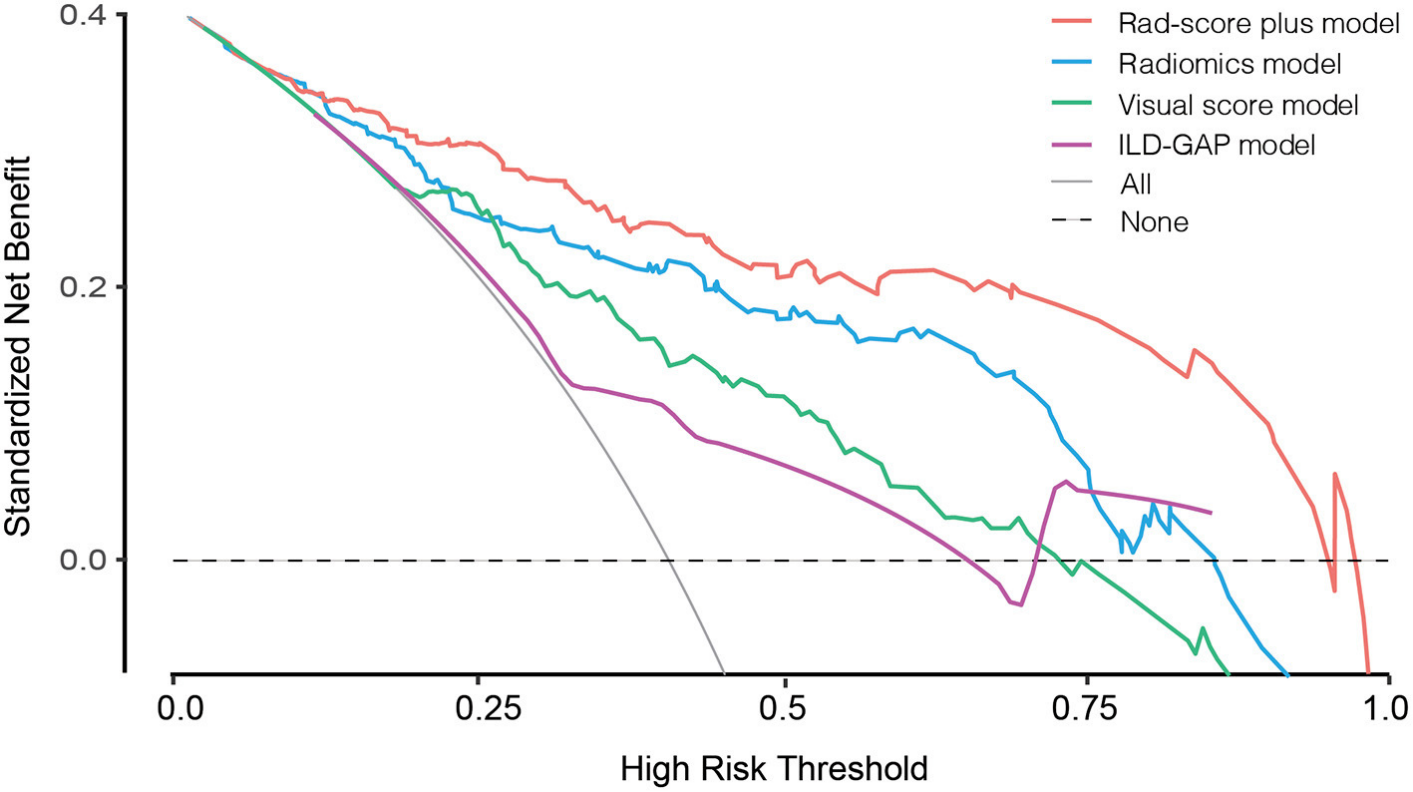
Calibration curves for the visual score model (green line), radiomics model (blue line), Rad-score plus model (red line), and ILD-GAP model (purple line). Calibration curves showed the calibration of each model in terms of the agreement between the predicted and observed 6-month outcomes. Nomogram-predicted outcome was plotted on the x-axis; the observed 6-month survival probability was plotted on the y-axis. Diagonal dotted line referred to perfect estimation by an ideal model, in which the predicted outcome perfectly corresponded to the actual outcome. Solid line referred to performance of the nomogram, a closer lining of which with the diagonal dotted line indicated better estimation.

**Figure 4:**
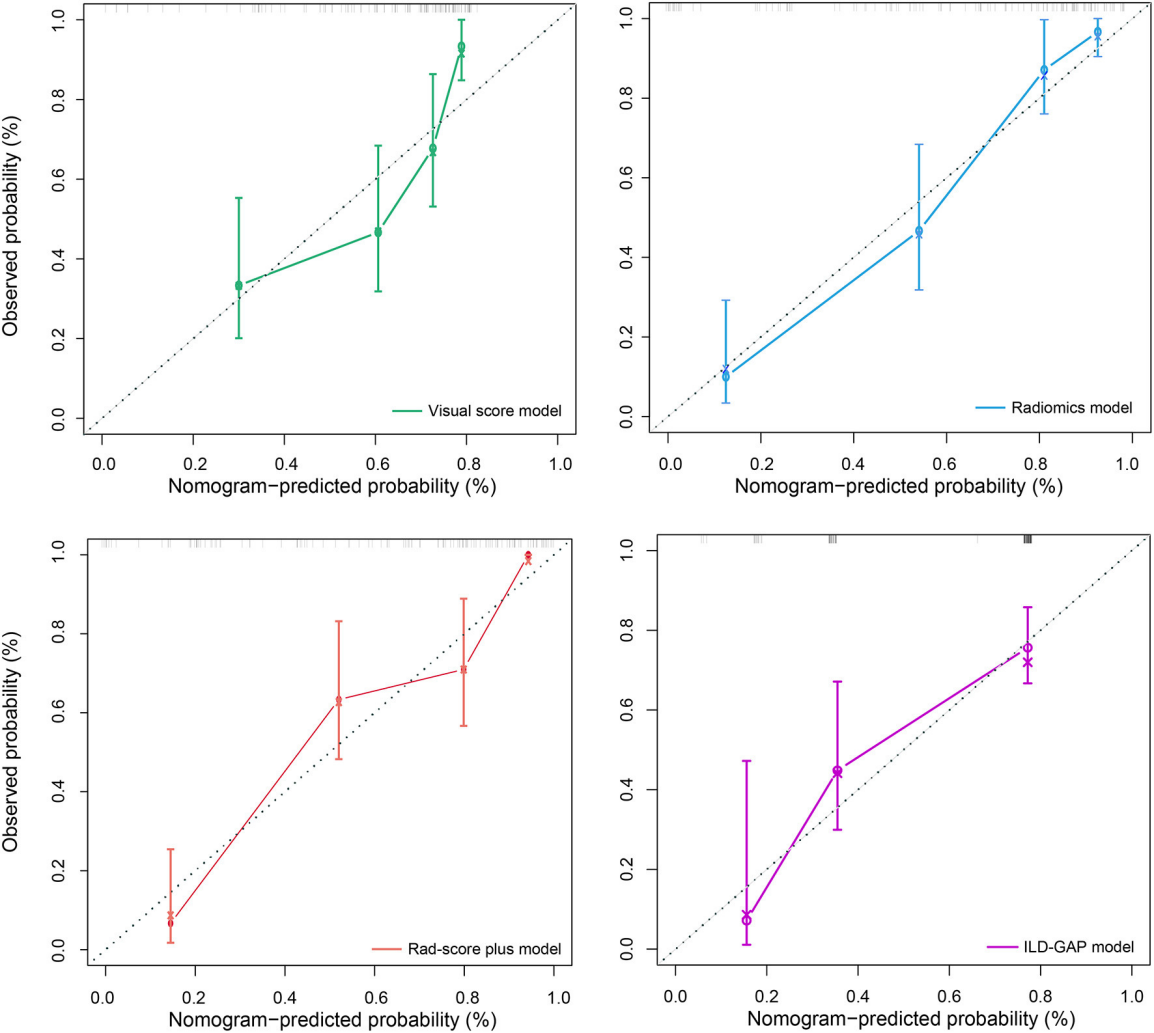
Decision curve analysis for ILD-GAP model, visual score model, Radiomics model, and Rad-score plus model. The concept of population net benefit (NB) is fundamental to decision curves (measured in the y-axis) and referred to classification accuracy of a model. Suppose high risk is defined as risk above some risk threshold R (x-axis), an intervention is recommended in such high-risk patients. The NB of using the risk model was calculated by the true-positive rate (TPRR), the proportion of cases with risk above risk threshold R, and the false-positive rate (FPRR), the proportion of controls with risk above risk threshold R. The horizontal dotted line at NB = 0 indicated a simple policy of no intervention to all patients (treat none); the gray curve in the plot depicted the NB of another simple policy: the intervention was recommended to everyone regardless of risk. In our result, the Rad-score plus model had the highest net benefit compared to others, almost across the full range of threshold probabilities.

The prediction accuracy and robustness of the ILD-GAP score, visual score, radiomics model, and Rad-score plus model are compared in Table 5, with respective calibration curves (Figure 3). The final Rad-score plus model had the best performance surpassing the radiomics model, visual score, and ILD-GAP score (*p* < 0.001). Finally, the decision curve analysis demonstrated that, in terms of clinical applicability, the Rad-score plus model also had a higher overall net benefit than other models across the majority of the ranges of threshold probabilities (Figure 4).

In the correlation analysis, the visual score, Rad-score, and Rad-score plus mentioned above all showed strong correlation with FVC and PaO_2_/FiO_2_ rather than other clinical predictors (for details, please see Supplementary Table 3).

### Subgroup Analysis

Across all datasets, radiomics performed better than visual score in 35 cases, which were defined as Rad-score better group. This subgroup presented a significantly higher mortality, a shorter disease course, a worse pulmonary function, and a higher dosage requirement of steroid (Supplementary Table 2), possibly implying that the radiomics model has a better performance in the patients with more progressive disease course.

## Discussion

In the current study, we first applied radiomics analysis in MDA5^+^ DM-ILD based on a large multicenter cohort. This radiomics approach could not only quantitatively assess the ILD on HRCT, but also excellently predict the prognosis of this tricky disease, surpassing the traditional visual score. Furthermore, the final Rad-score plus model, integrating Rad-score and clinical parameters, showed a superior discriminative performance.

MDA5^+^ DM-ILD remains to be a great challenge for us despite early diagnosis and recent treatment advances (4, 24). Immunosuppressive therapy has been widely accepted as the cornerstone of treatment for this tricky disease. However, the therapeutic effect of a specific regimen has not been demonstrated depending on risk or severity stratification. Accumulative prognostic factors of the disease have been reported involving aspects of respiratory physiology, laboratory markers, and radiology (2, 5, 7, 25). To the best of our knowledge, the current study is the first attempt to quantitatively assess MDA5^+^ DM-ILD by applying the computer-based radiomics approach, which enriches the panorama of prognostic prediction for this disease.

Our data illustrated that the Rad-score derived from multiple radiomic features could provide a reliable predictor for the 6-month mortality of MDA5^+^ DM-ILD, and outperform the visual CT score. It implied that radiomics enhanced the quantification of chest CT images as it provided more comprehensive features to describe the distribution, heterogeneity of CT value, and texture information behind the visual image. Shape and texture features were the predominant components of the Rad-score. The selected shape feature of flatness, which indicated the change in the lung shape during the progression of the disease, had a negative impact on prognosis. The texture features of MCC, IDMN, and Large AreaHighGrayLevelEmphasis, describing the complexity of texture or the distribution of gray level value, implied that the survival outcome was worse with the increasing heterogeneity of the texture. Such heterogeneity might impute to the lesions of GGA, consolidation, and reticulation pattern, which were reported by Hozumi et al. as the most distinctive HRCT patterns for MDA5^+^ DM-ILD (26). Skewness measured the asymmetry of CT value distribution, which was also discovered to indicate the disease progression of IPF in previous studies (27). Of note, in the population of MDA5^+^ DM-ILD, the organizing pneumonia pattern was found to be dominant in HRCT, with lower zone consolidation correlating with rapidly progressive ILD (28). Moreover, another study with pathological evidences discovered that a perilobular opacity transforming to consolidation corresponded to diffuse alveolar damage, which was presented as the main finding in MDA5^+^ DM associated with rapidly progressive ILD (29). Thus, these manifestations might result in the changes of CT values and texture, making these radiomic features important for diagnosis, risk stratification, and prognosis. In addition, the Rad-score performed even better than visual score in a more aggressive subgroup with worse prognosis, supported by our subgroup analysis.

Consistent with our previous study of prognosis on this disease, the clinically relevant parameters were age and FVC% levels in the final Rad-score plus model, and coincidentally, they were also the main components in GAP staging system (18, 30). Moreover, HRCT imaging in combination with pulmonary function test was acknowledged as the gold standard for a pivotal and non-invasive assessment of ILD (31). Thus, it was rational to combine FVC% with Rad-score to further enhance its predictive performance.

The limitations of our study first lie in the retrospective design of data collection and relatively limited sample size of external validation. Notably, the treatment regimens were relatively dissimilar between two centers, with significantly higher intensity of glucocorticoid and immunosuppressant exposure in the external validation cohort. However, the overall mortality and other baseline parameters were comparable between them. The verification of our findings would be better in a larger-scale prospective multicenter validation cohort, by which the biases of different machine conditions, patient selection, and treatment protocols could be minimized and controlled. However, even in tertiary centers, the rarity of this disease is well recognized. Second, since the computer-based radiomic features are known as agnostic features (12) and beyond visual conception, it is difficult to interpret the Rad-score pathophysiologically. Possibly, further work in radiogenomics may help to establish an in-depth link between radiomic changes and pathogenesis. Lastly, the feature extraction was based on the whole lung rather than the visible extent of ILD. Although this could include all information on the lung, it may also dilute the texture features of the affected regions. Further research focused on the affected regions is required.

## Conclusions

A quantitative computer-derived radiomic signature was a superior and reliable prognostic predictor for the 6-month mortality in MDA5^+^ DM-ILD. This instrument may grant a step forward to elegant clinical trial design and precision management of this challenging disease.

## Data Availability Statement

The datasets presented in this study can be found in online repositories. The names of the repository/repositories and accession number(s) can be found in the article/Supplementary Material.

## Ethics Statement

Written informed consent was obtained from the individual(s) for the publication of any potentially identifiable images or data included in this article.

## Author Contributions

WX, WW, QH, YZho, and SY contributed to the project design. WX, WW, ZC, and DZ collected the clinical data of derivation cohort. BG, QL, and QH collected the clinical data of validation cohort. JZ and KW were responsible for laboratory data. YZhe contributed to the assessment of pulmonary function tests. ZC and YZhe conducted visual HRCT evaluation. WX, YZhe, and XT operated the Radiomics software. WX, WW, and XT contributed to analysis of all data. WX, WW, and SY wrote and revised the manuscript. All authors have read and agreed to the final version of the manuscript.

## Funding

This study was supported by Clinical Research Plan of SHDC (No. SHDC2020CR1015B).

## Conflict of Interest

XT is an employee of Siemens Healthineers. The remaining authors declare that the research was conducted in the absence of any commercial or financial relationships that could be construed as a potential conflict of interest.

## Publisher's Note

All claims expressed in this article are solely those of the authors and do not necessarily represent those of their affiliated organizations, or those of the publisher, the editors and the reviewers. Any product that may be evaluated in this article, or claim that may be made by its manufacturer, is not guaranteed or endorsed by the publisher.
