# Supplementary material for: A Computed Tomography Radiomics-Based Prediction Model on Interstitial Lung Disease in Anti-MDA5-Positive Dermatomyositis
Source: Front Med (Lausanne). 2021 Nov 29;8:768052. doi: 10.3389/fmed.2021.768052 (PMC8667862; doi:10.3389/fmed.2021.768052)
Supplement: Supplementary file 2 [file Table_2.docx]

| Supplementary table 2: Comparison of Rad-score better group and the rest of all datasets. | | | | |  |
| --- | --- | --- | --- | --- | --- |
| **Characteristics** | All datasets  (n=228) | Rad-score better group (n=35) | The rest of group (n=193) | P-value |  |
| Six-month mortality | 93 (40.8%) | 26 (74.3%) | 67 (34.7%) | <0.001 |  |
| **Demographic** |  |  |  |  |  |
| Male sex | 85 (37.3%) | 17 (48.6%) | 68 (35.2%) | 0.19 |  |
| Age, years | 51 [43-57] | 53 [45.5-64] | 50 [43-56] | 0.018 |  |
| DM course*, month | 2 [2-4] | 2 [1-3] | 2 [2-4] | 0.043 |  |
| ILD-course**^†^**, week | 4 [2-8] | 4 [2-5.5] | 4 [2-8] | 0.067 |  |
| **Extrapulmonary symptoms** |  |  |  |  |  |
| Fever | 126 (64.3%) | 22 (73.3%) | 104 (62.7%) | 0.359 |  |
| Arthralgia | 96 (49.0%) | 8 (26.7%) | 88 (53.0%) | 0.014 |  |
| Pharyngalgia | 28 (14.3%) | 2 (6.67%) | 26 (15.7%) | 0.263 |  |
| Heliotrope sign | 171 (87.2%) | 27 (90.0%) | 144 (86.7%) | 0.773 |  |
| Gottron sign | 162 (82.7%) | 25 (83.3%) | 137 (82.5%) | 1 |  |
| Skin ulcer | 35 (17.9%) | 3 (10.0%) | 32 (19.3%) | 0.036 |  |
| Dysphagia | 28 (14.3%) | 3 (10.0%) | 25 (15.1%) | 0.581 |  |
| **Pulmonary function** |  |  |  |  |  |
| FVC% | 62.8 (20.2) | 55.3(18.7) | 64.0(20.2) | 0.05 |  |
| Unable to perform PFT**^‡^** | 127 (57.5%) | 13 (38.2%) | 114 (61.0%) | 0.8 |  |
| PaO_2_/FiO_2_, mmHg | 338 [262-402] | 267 [239-308] | 357 [280-405] | 0.82 |  |
| **Laboratory data** |  |  |  |  |  |
| Serum ferritin, ng/mL | 1480 [661-2676] | 2336 [1365-4856] | 1415 [623-2528] | 0.009 |  |
| LDH, U/L | 302 [240-434] | 342 [268-434] | 298 [238-434] | 0.441 |  |
| CRP, mg/L | 3.89 [0.02-11.8] | 4.08 [0.00-25.1] | 3.89 [0.10-10.3] | 0.611 |  |
| ESR, mm/H | 31.0 [16.0-46.5] | 34.0 [14.8-64.0] | 30.0 [16.0-44.0] | 0.31 |  |
| Lymphocyte, 10^9/L | 0.71 [0.43-1.00] | 0.60 [0.41-0.76] | 0.73 [0.49-1.04] | 0.065 |  |
| Cytopenia | 40 (17.5%) | 7 (20.0%) | 33 (17.1%) | 0.862 |  |
| CKmax, U/L | 98 [38-233] | 101 [50.0-274] | 89.0 [37.0-222] | 0.186 |  |
| ALT, U/L | 57 [35.0-103] | 74.0 [49.0-158] | 54.0 [34.0-95.0] | 0.017 |  |
| AST, U/L | 55.1 [36.0-98.5] | 65.0 [41.0-202] | 54.0 [35.3-92.0] | 0.085 |  |
| Anti-Ro52 Ab positive | 148 (64.9%) | 27 (77.1%) | 121 (62.7%) | 0.146 |  |
| Anti-MDA5 Ab titer, RU/mL | 187 (53.9%) | 199 (55.8) | 185 (53.5) | 0.164 |  |
| **Treatment** |  |  |  |  |  |
| Max dosage of MP, mg/d | 120 [80-240] | 160 [120-245] | 80.0 [80.0-240] | 0.013 |  |
| Steroid pulse therapy | 37 (16.2%) | 7 (20.0%) | 30 (15.5%) | 0.683 |  |
| Exposure to IS**^§^** |  |  |  |  |  |
| 1 IS | 83 (36.6%) | 9 (25.7%) | 74 (38.5%) | 0.196 |  |
| >=2 IS | 130 (57.02%) | 23 (65.7%) | 107 (55.4%) | 0.345 |  |
| Exposure to pirfenidone or nintedanib | 99 (43.4%) | 20 (57.1%) | 79 (40.9%) | 0.111 |  |
| Data are presented as median [IQR] for continuous variables and number (frequency) (%) for categorical variables. | | | | |  |
| *DM course, time from the first symptom of dermatomyositis (DM) to admission  †ILD-course, time from the first abnormal pulmonary CT that revealed ILD changes to admission  ‡Unable to perform PFT, referred to those patients with severe condition who were unable to complete either routine or bedside spirometry  §IS, immunosuppressant drugs, include cyclophosphamide, cyclosporine, tacrolimus, mycophenolate mofetil, tofacitinib, rituximab, basiliximab, and tocilizumab. ILD, interstitial lung disease; FVC%, forced vital capacity percentage of predicted; PFT, pulmonary function test; PaO2/FiO2, arterial oxygen/fraction of inspiration oxygen; CRP, C-reactive protein; ESR, erythrocyte sedimentation rate; LDH, lactate dehydrogenase; CKmax, maximum creatine kinase from disease onset to admission; ALT, alanine transaminase; AST, aspartate transaminase; MDA5, melanoma differentiation-associated protein 5; Ab, antibody; MP, methylprednisolone. | | | | |  |
|  |  |  |  |  |  |
|  |  |  |  |  |  |
|  |  |  |  |  |  |
|  |  |  |  |  |  |
|  |  |  |  |  |  |
