# Supplementary material for: A Computed Tomography Radiomics-Based Prediction Model on Interstitial Lung Disease in Anti-MDA5-Positive Dermatomyositis
Source: Front Med (Lausanne). 2021 Nov 29;8:768052. doi: 10.3389/fmed.2021.768052 (PMC8667862; doi:10.3389/fmed.2021.768052)
Supplement: Supplementary file 3 [file Table_3.docx]

Supplementary table 3: Correlation analysis between visual score, Rad-score, Rad-score plus and partial clinical parameters.

| Correlation analysis | Visual score | |  | Rad-score | |  | Rad-score plus | |
| --- | --- | --- | --- | --- | --- | --- | --- | --- |
|  | Spearman  (ρ) | p-value |  | Spearman  (ρ) | p-value |  | Spearman  (ρ) | p-value |
| Age at admission | 0.17 | 0.01 |  | 0.41 | <0.001 |  | 0.53 | <0.001 |
| DM course* | 0.11 | 0.09 |  | 0 | 0.99 |  | -0.02 | 0.8 |
| FVC% | -0.59 | <0.001 |  | -0.48 | <0.001 |  | -0.44 | <0.001 |
| three-category FVC% | 0.62 | <0.001 |  | 0.54 | <0.001 |  | 0.5 | <0.001 |
| PaO_2_/FiO_2_ | -0.72 | <0.001 |  | -0.68 | <0.001 |  | -0.65 | <0.001 |
| Serum ferritin | 0.09 | 0.2 |  | 0.25 | <0.001 |  | 0.26 | <0.001 |
| C-reactive protein | 0.2 | 0.004 |  | 0.26 | <0.001 |  | 0.26 | <0.001 |
| LDH | 0.33 | <0.001 |  | 0.3 | <0.001 |  | 0.28 | <0.001 |
| Lymphocyte | -0.27 | <0.001 |  | -0.29 | <0.001 |  | -0.3 | <0.001 |
| Anti-MDA5 Ab titer | 0.01 | 0.85 |  | 0.1 | 0.15 |  | 0.11 | 0.09 |
| Rad-score | 0.69 | <0.001 |  | / | / |  | / | / |
| Visual score | / | / |  | 0.69 | <0.001 |  | / | / |

*DM course, time from the first symptom of dermatomyositis (DM) to admission;

FVC%, forced vital capacity percentage of predicted; PaO2/FiO2, arterial oxygen/fraction of inspiration oxygen; LDH, lactate dehydrogenase; MDA5, melanoma differentiation-associated protein 5; Ab, antibody.
