# Supplementary material for: A Computed Tomography Radiomics-Based Prediction Model on Interstitial Lung Disease in Anti-MDA5-Positive Dermatomyositis
Source: Front Med (Lausanne). 2021 Nov 29;8:768052. doi: 10.3389/fmed.2021.768052 (PMC8667862; doi:10.3389/fmed.2021.768052)
Supplement: Supplementary file 4 [file Data_Sheet_1.docx]

**Supplementary material**

**Materials and methods**

**Measurement of autoimmune antibodies**

The semiquantitative detection of anti-MDA5 and other myositis specific antibodies (MSAs) was performed using EUROLINE Autoimmune Inflammatory Myopathies 16 Ag (IgG) (Euroimmun, Germany).

Quantification of anti-MDA5 antibody as confirmatory was conducted by enzyme-linked immunosorbent assay (ELISA). First, purified recombinant MDA5 antigen (rMDA5) (Freezone Biotechnology co., LTD, Shanghai, China) diluted to 5 μg/mL in phosphate-buffered saline (PBS) was coated onto 96-well Microtiter plates (Maxisorp; Nunc, Rochester, NY, USA) overnight at 4°C. The plates were washed twice with PBS and blocked with PBS containing 1% bovine serum albumin (BSA) and 5% sucrose overnight at 4°C. Second, the serum samples were diluted at 1:101 in PBS containing 0.5% sodium chloride, 0.15% Tween 20, 0.2% BSA and incubated for 30 min at room temperature. Then, the plates were washed four times with PBS containing 0.05% Tween 20 and incubated with Goat-conjugated anti-human IgG (PROMEGA, USA) diluted 1:60000 in conjugate stabilizer (Thermo, USA). Finally, after incubation for 30 min at room temperature, the plates were washed four times and the bound antibodies were detected with the peroxidase substrate, 3, 3’, 5, 5’-tetramethylbenzidine. After incubation for 10 min at room temperature, the reaction was stopped by the addition of 0.5 N sulfuric acid. Absorbance at 450 nm (A) was measured, and unit values (IU/mL) were calculated from the following formula: 100 × (sample OD - blank OD) / (anti-MDA5-positive reference OD - blank OD). The cutoff level was set at 35 IU/mL.
